# An unusual case of ectopic corticotrophin-releasing hormone syndrome caused by an adrenal noncatecholamine-secreting pheochromocytoma: a case report

**DOI:** 10.1186/s12902-018-0269-8

**Published:** 2018-06-19

**Authors:** Bao-Ping Wang, Lei-Lei Yang, Hao Wang, Qing He, Zhong-Shu Ma, Yi Lin, Chang-Xin Jiang, Hao-Ran Sun, Ming Liu

**Affiliations:** 1 Department of Endocrinology and Metabolism, Tianjin Medical University General Hospital, No 154 Anshan Road, Heping District, Tianjin, 300052 China; 2 Department of Gerontology, Beijing ChuiYangLiu Hospital (Chui Yang Liu Hospital affiliated to TsingHua University), Beijing, 100022 China; 3 Department of Urology, Tianjin Medical University General Hospital, No 154 Anshan Road, Heping District, Tianjin, 300052 China; 4 Department of Pathology, Tianjin Medical University General Hospital, No 154 Anshan Road, Heping District, Tianjin, 300052 China; 5 Department of Medical Imaging, Tianjin Medical University General Hospital, No 154 Anshan Road, Heping District, Tianjin, 300052 China

**Keywords:** Adrenocorticotropin, Dexamethasone, Ectopic CRH syndrome, Pheochromocytoma, Noncatecholamine secreting

## Abstract

**Background:**

Pheochromocytoma, especially for noncatecholamine-secreting pheochromocytoma, is an extremely rare cause of ectopic corticotrophin-releasing hormone (CRH) syndrome.

**Case presentation:**

A 27-year-old Chinese woman was administered dexamethasone for a skin allergy, but her general condition rapidly deteriorated over a month. She was subsequently hospitalized for typical clinical features of Cushing’s syndrome. Endocrinological investigation confirmed severe hypercortisolism along with elevated plasma adrenocorticotropin hormone (ACTH). However, magnetic resonance imaging (MRI) revealed no pituitary adenoma. Abdominal contrast-enhanced computed tomography (CT) revealed a 6.5 cm heterogeneous right adrenal mass with mildly contrast enhancement. The tumor was found during a routine physical check-up at a local hospital 16 months ago; however, the patient did not have any symptoms and did not seek further medical attention at that time. Laparoscopic resection of the right adrenal tumor led to a rapid remission of Cushing’s syndrome. Based on pathological findings and the presence of normal catecholamine metabolites in her serum and urine, the patient was diagnosed with noncatecholamine-secreting pheochromocytoma. Immunohistochemical staining of the adrenal tumor revealed positive staining for CRH and negative staining for ACTH.

**Conclusions:**

This is an extremely rare case of ectopic CRH syndrome caused by an adrenal noncatecholamine-secreting pheochromocytoma. Both ectopic ACTH syndrome and ectopic CRH syndrome should be considered in patients presenting with ACTH-dependent Cushing’s syndrome caused by extrapituitary diseases.

## Background

Cushing’s syndrome is classified as either ACTH-independent or ACTH-dependent, which can be further classified as either Cushing’s disease or ectopic ACTH syndrome (EAS). EAS accounts for 10–20% of all cases of Cushing’s syndrome [1]. The most common origins of the tumors responsible for EAS are the lungs (45%), thymus (11%), pancreas (8%), and thyroid (6%) [2]. Pheochromocytoma accounts for approximately 5% of EAS cases [2]. Approximately 1.3% of all identified pheochromocytomas have ectopic ACTH secretion [3]; in rare cases, the ectopically secreted hormone is CRH, with ACTH being secreted by the pituitary gland [4]. To date, only one case has been reported where a patient with an ectopic CRH-secreting pheochromocytoma had normal catecholamine metabolites [5]. Dexamethasone has negative feedback on CRH gene expression and secretion in the hypothalamus. However, dexamethasone could stimulate CRH expression in the placenta and the bed nucleus of the stria terminalis, suggesting that dexamethasone regulation might be tissue-specific [6]. Here, we reported a patient with ectopic CRH syndrome caused by an adrenal noncatecholamine-secreting pheochromocytoma that was associated with a use of dexamethasone.

## Case presentation

Prior to this admission, a 27-year-old woman sought medical attention at a local hospital because of facial redness and edema caused by eating a mango. She was treated with dexamethasone (5 mg intravenously daily) for five days. The patient gradually developed a round face, acne, hirsutism, hypokalemia, and 5 kg of weight loss over the course of one month since receiving dexamethasone. Upon hospitalization, the patient presented with a one-month history of facial edema, weight loss, and acne. She had no family history of Cushing’s syndrome, pheochromocytoma, or multiple endocrine neoplasia type 2. A 5.5-cm mass located in the right posterior lobe of the liver was detected by ultrasound in a routine physical examination 16 months ago at a local hospital; however, the patient had no symptom and did not seek further medical attention at that time.

The patient’s blood pressure was 120/75 mmHg in both arms in the supine position, with a regular pulse of 76 bpm. Her height was 164 cm and her weight was 48 kg (body mass index:17.8 kg/m^2^). The patient had “moon face” and severe facial edema, beard, central deposition of fat with slim extremities and atrophic muscles, and no pretibial edema. The skin was diffused with acne and both armpits had hyperpigmentaion.

Laboratory tests revealed marked hypokalemia (2.1 mmol/L; normal range, 3.5–5.5 mmol/L), which could not be normalized with oral and intravenous potassium supplementation until spironolactone was added. A 75-g oral glucose tolerance test confirmed diabetes mellitus with a fasting blood glucose level of 9.19 mmol/l and a 2-h glucose level of 21.66 mmol/L, with a HbA1c level of 6.3%. She was started on insulin aspart30 (48 U daily).

Endocrinological investigation identified severe hypercortisolism with loss of circadian rhythm. Plasma ACTH level was elevated to 1157 pg/mL, confirming ACTH-dependent Cushing’s syndrome. Except for testosterone, the catecholamine metabolites, growth hormone, calcitonin, and prolactin levels were all within the normal range (Table 1).

**Table 1:** Laboratory findings of pertinent hormones

	Baseline value	Overnight dexamethasone suppression test	Normal range
Serum cortisol			5–25 μg/dL
08:00 h	>50	>50	
16:00 h	>50		
00:00 h	>50		
Urine free cortisol	>2000		30–110 μg/24 h
ACTH			0–46 pg/mL
08:00 h	1157	625	
16:00 h	459		
00:00 h	350		
VMA	20.5		<72 μmol/24 h
Metanephrine	0.15		≤0.5 nmol/L
Normetanephrine	0.13		≤0.9 nmol/L
FSH	5.36		2.5–10.2 IU/L
LH	0.4		1.9–12.5 IU/L
GH	0.12		0.06–5 ng/mL
PRL	0.3		2.8–29.2 ng/mL
T	154.9		14–76 ng/dL
E2	26.18		19–144 pg/mL
CT	<2		0–5 pg/mL
TSH	0.014		0.3–5.0 IU/mL
FT4	11.94		11.5–23.5 ng/dL
FT3	2.21		3.5–5.5 ng/dL
rT3	0.34		0.2–0.4 ng/mL

VMA: 24-hurinary vanillylmandelicacid; FSH: follicle-stimulating hormone; LH: luteinizing hormone; E2: estradiol; CT: calcitonin; PRL: prolactin; T: testosterone; FT3: free thyroxin; FT4: free triiodothyronine; TSH: thyroid stimulating hormone; rT3: reverse thyroxin

**Fig. 1:**
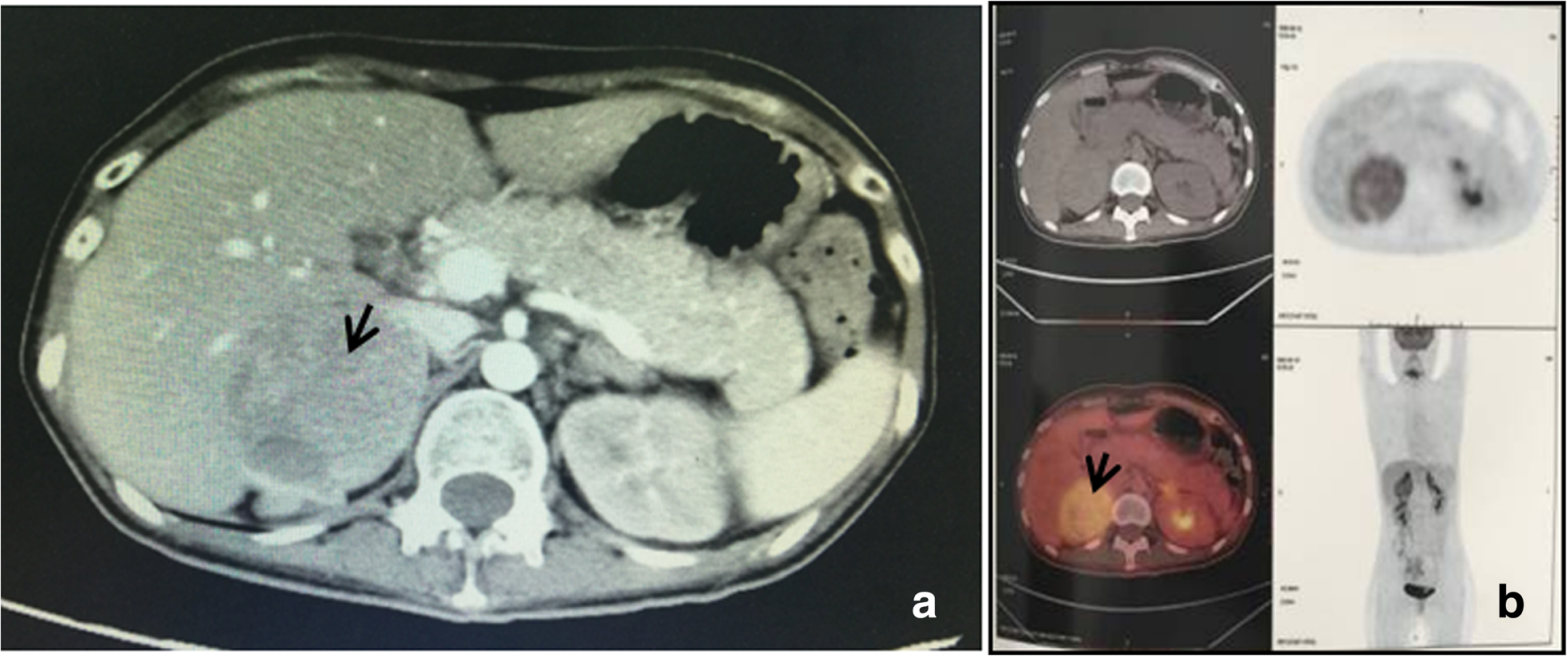
The CT and PET/CT image of the adrenal pheochromocytoma. **a** Abdominal contrast-enhanced CT revealing a mass with a diameter 6.5 cm in the right adrenal gland. The mass was a heterogeneous solid tumor which was mildly enhanced with some patchy nonenhancing areas (arrow). **b** PET/CT showed moderate FDG uptake in the right adrenal mass(arrow)

MRI and contrasted MRI revealed no pituitary adenoma and EAS was considered. Abdominal contrast-enhanced CT scanning revealed that the mass lesion was located in the right adrenal gland and not in the right hepatic posterior lobe. The mass was a heterogeneous solid tumor which was mildly enhanced with some patchy nonenhancing areas (Fig. 1a). PET/CT showed moderate fluorodeoxyglucose (FDG) uptake in the mass (Fig. 1b). Both contrasted CT and PET-CT revealed bilateral adrenal hyperplasia, but the neck, thorax, and pelvis were normal.

**Fig. 2:**
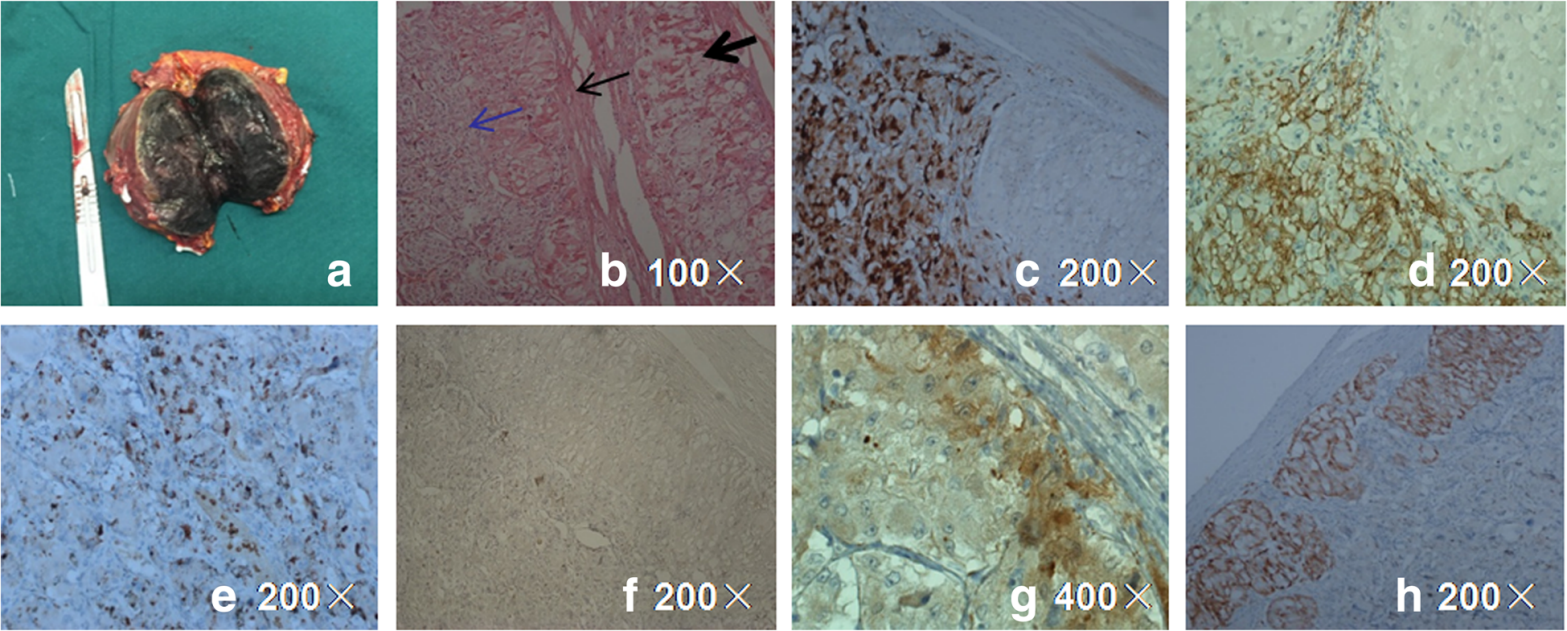
External appearance and histopathology of the pheochromocytoma. **a** External appearance of the resected tumor, 6.5 cm in diameter and black in color, from the medial branch of right adrenal gland with enlargement of the lateral branch. **b** Histopathology revealed that the majority of cells were chromaffin-like cells with a very rich vascular sinus (thin blue arrow). Beneath the tumor capsule, there were multifocal oval eosinophilic cells with oval nucleus (thin black arrow). Adrenocortical hyperplasia was also revealed (bold black arrow; HE staining, 100×). Positive immunohistostaining is shown for CgA (**c** 200×), CD56 (**d** 200×), and Ki67 (**e** 100×) for chromaffin-like cells. No positive ACTH immunostaining was noticed (**f** 200×). Positive immunohistostaining is shown for CRH (**g** 400×) and Melan-A (**h** 200×) in the eosinophilic cells

We prepared to do inferior petrosal sinus sampling (IPSS) for ACTH assays, adrenal vein sampling for ACTH assays to make out the origin of ACTH. But the patient’s general condition rapidly deteriorated after admission, Adrenal tumorectomy was performed. During surgery, when the right adrenal mass was mobilized, no hypertensive crisis occurred. A 6.5-cm black mass was found arising from the medial branch of the right adrenal gland (Fig. 2a). Hematoxylin-eosin staining of the tumor revealed that most of the cells were chromaffin-like cells. In addition, there were multifocal oval eosinophilic cells under the tumor capsule (Fig. 2b). Immunohistochemical staining showed positive staining for chromogranin A (CgA) (Fig. 2c) and CD56 (Fig. 2d), with a Ki67 labeling index of approximately 16% (Fig. 2e) for chromaffin-like cells. No positive ACTH immunostaining was noticed (Fig. 2f). Positive immunostaining for CRH and Melan-A (Fig. 2g and h) and negative immunostaining for CgA and CD56 were found in the eosinophilic cells, indicating that CRH production is indeed derived from the tumor’s peripheral cells.

One day after surgery, ACTH levels had decreased from 715 to 14.3 pg/ml and serum cortisol level had decreased from more than 50 to 10.4 μg/dl. One week postoperatively, blood potassium and glucose levels normalized without the need for medication. The signs and symptoms of Cushing’s syndrome gradually disappeared within two months, and the hydrocortisone supplementation (initial dosage was 60 mg daily and tapered gradually) was discontinued seven weeks postoperatively. CT revealed that the left adrenal gland was almost reduced to the normal size three months later. The patient is currently under regular follow-up and remains well nine months after surgery.

## Discussion

EAS is rare, with approximately 5% of all cases caused by pheochromocytoma [2]. In our patient, a diagnosis of EAS was suggested by the relatively short history, severe Cushing’s syndrome associated with refractory hypokalemia, the absence of a definitive lesion on the pituitary MRI scan, and the extremely elevated plasma ACTH levels. Histology confirmed that the right adrenal mass was a pheochromocytoma. The clinical features and the presence of normal catecholamine metabolites in the patient’s serum and urine confirmed the presence of a noncatecholamine-secreting pheochromocytoma. Chen et al. proposed criteria for the diagnosis of ACTH-secreting pheochromocytomas [7]. Except for the biochemical evidence of pheochromocytoma, this case satisfied all criteria for ACTH-secreting pheochromocytoma.

Some CRH-secreting tumors (pheochromocytoma in this case) could be misdiagnosed as ACTH-secreting tumors because preoperative inferior petrosal sinus sampling (IPSS) for ACTH assays, adrenal vein sampling for ACTH and CRH assays, and postoperative immunostaining for ACTH and CRH are not routinely performed. Quinton et al. reported three patients with ACTH-dependent Cushing’s syndrome; in two cases, immunostaining showed reactivity for CRH instead of ACTH, supporting CRH (or related peptide) -secreting pheochromocytoma [8]. In the current case, the patient’s rapidly deteriorating condition did not allow us to perform IPSS and adrenal vein sampling. Yet, negative immunostaining for ACTH and positive immunostaining for CRH strongly suggested that the elevated ACTH resulted from an adrenal CRH-secreting pheochromocytoma.

Studies have reported that EAS and ectopic CRH syndrome have different effects on the hypothalamic-pituitary-adrenal axis [8]. Post-operative recovery from the clinical symptoms and elevated cortisol levels is much faster in patients with ectopic CRH syndrome than in those with ectopic ACTH syndrome [5, 8]. In the present case, the rapid postoperative recovery of clinical features, elevated serum ACTH, potassium and glucose levels, and short-term application of prednisone supported the classification of the pheochromocytoma as a CRH-secreting tumor, with ACTH secreted from the pituitary gland. Pathological evidence, clinical presentations, and outcomes supported the ectopic CRH syndrome diagnosis.

An ectopic CRH-secreting pheochromocytoma is an extremely rare cause of ACTH-dependent Cushing’s syndrome. The first case of isolated ectopic CRH-secreting pheochromocytoma was reported in 1999 [4]. To date, six cases of isolated ectopic CRH-secreting pheochromocytoma and three cases of ectopic ACTH/CRH co-secreting pheochromocytoma have been reported [4, 5, 8–13]. Usually, a pheochromocytoma produces catecholamines and causes symptoms such as hypertension [14]. Among these six cases of isolated CRH-secreting pheochromocytoma, only one case had normal plasma and urinary epinephrine, normetanephrine, and metanephrine concentrations [5]. Although there is no record of hypertensive crisis during surgery, the event cannot be completely ruled out for functional pheochromocytoma because the patient was treated preoperatively with the somatostatin analog octreotide [5]. In the current case, the lack of catecholamine hypersecretion, the clinical manifestations, and the consistently normal blood and urinary levels of catecholamines and their metabolites provided no indication of a pheochromocytoma. To our knowledge, this is the second reported case of Cushing’s syndrome caused by ectopic CRH secreted from an adrenal noncatecholamine-secreting pheochromocytoma.

In this case, the patient had an adrenal tumor without any symptoms for 16 months before this hospitalization. The typical clinical features of Cushing’s syndrome appeared to be associated with administration of dexamethasone that was used to treat a skin allergy caused by eating mango. To our knowledge, mangoes or allergies have no effect on CRH synthesis and secretion, but this is not true for dexamethasone. Dexamethasone has negative feedback on hypothalamus CRH gene expression and secretion, but tissue-specific regulation of dexamethasone on CRH gene expression and secretion may exist. Indeed, glucocorticoids could stimulate CRH synthesis and secretion in the human placenta [6], and dexamethasone could up-regulate CRH gene expression in the pheochromocytoma [13]. In this case, a pre-existing tumor, chronological dexamethasone administration, and clinical symptoms and signs suggested that dexamethasone might induce CRH gene expression and/or secretion in the adrenal pheochromocytoma, thereby may provoke ectopic CRH syndrome. However, due to lack of blood tests before onset of symptom, we cannot rule out the possibility that the patient might have mild hypercortisolism before dexamethasone administration. Nevertheless, the current case suggests that performing dexamethasone suppression test may need to be evaluated if ectopic CRH syndrome is considered.

This study has several limitations. First, owing to the lack of a CRH assay, plasma CRH levels were not measured. Therefore, there was no direct evidence of elevated plasma CRH levels in this patient. Second, because the patient’s condition rapidly deteriorated, pre-surgery IPSS and adrenal vein sampling for measuring ACTH were not performed, which could have provided direct evidence of the source of elevated ACTH. Finally, dexamethasone’s effect on the onset and development of ectopic CRH syndromes was postulated based on the chronology of dexamethasone administration and presentation of clinical features and syndromes. Further experimental studies are warranted to confirm the tissue-specific regulation of dexamethasone on CRH expression and secretion.

## Conclusions

In conclusion, we report an extremely rare case of ectopic CRH syndrome caused by an adrenal noncatecholamine-secreting pheochromocytoma. Both ectopic ACTH syndrome and ectopic CRH syndrome should be considered in patients presenting with ACTH-dependent Cushing’s syndrome caused by extrapituitary diseases.

## Abbreviations

ACTH: Adrenocorticotropin hormone
CRH: Corticotrophin-releasing hormone
CT: Computed tomography
EAS: Ectopic ACTH syndrome
FDG: Fluorodeoxyglucose
IPSS: Inferior petrosal sinus sampling
MRI: Magnetic resonance imaging
PET: Positron emission tomography
